# 

**Published:** 1842-09

**Authors:** 


### CONTENTS

OF NO. III.

---

### ORIGINAL COMMUNICATIONS.

### ESSAYS AND CASES.

Art. I.—Observations on the epidemic Yellow Fever of Natchez, and of the South-west. By John W. Monette, M. D., of Washington, Mississippi. - - 161

Art. II.—Facts and Conjectures on the Trembles and Milk-sickness. In a letter from Dr. G. B. Taylor, of Morganfield, Kentucky, to Dr. W. L. Sutton, of Georgetown in this State; communicated by the latter with some remarks. - - - - 181

Art. III.—Wound of the Antrum of Highmore, with destruction of the eye; fragment of knife-blade removed from the antrum two years afterwards. By W. H. Donne, M. D., of Louisville. Reported by R. S. Wendel, Student of Medicine. - - - - 186

### SELECTIONS FROM AMERICAN AND FOREIGN JOURNALS.

Effects of the Times on the Profession. - - - 189

Scrivener's Spasm cured by Division of Muscles. - 190

Complete Prolapsus and Separation of the Vagina. - 191

Injury to the Cartilages of the Ribs—Removal of a portion of them, and Cure. - - - - - 192

Influence of the Nerves on Muscular Irritability. - - 193

New Mode of Treating Hydrocele. - - - 194
First Introduction of Syphilis into Scotland. - - 196
Clinical Lecture on Delirium Tremens. - - 196
Experiments on Kiesteine, with observations on its application to the diagnosis of pregnancy. - - - 206
Dentition of Children at the Breast. - - - 215
Expulsion of a Mass of Hair from the Uterus. - - 217
Structure of the Small-pox Pustule. - - - 219
Permanent Contraction of the Fingers. - - - 223
New Theory of Tinea; its supposed Vegetable Origin. - 228
Case of Gun-shot Wound of the Face. - - . 230
Account of a Phrenological Visit to the Penitentiary for young Criminals. - - - - - 233

ORIGINAL INTELLIGENCE.

Report on the Medical College. - - - 235
University of Virginia.—Latin Diplomas. - - 236
Dunglison's and Bell's Medical Libraries. - - 238
Private Medical Instruction. - - - - 239
Medical Appointments. - - - - 239
Gross' Edition of Liston's Elements of Surgery. - 240
Books received. - - - - - 240

